# Prevalence and Antimicrobial Resistance Profiles of Enterococci Isolated From Humans, Animals, and Environment in Bangladesh: A Systematic Review and Meta‐Analysis

**DOI:** 10.1155/ijm/6574310

**Published:** 2026-09-23

**Authors:** Md. Abdur Nur Sakib, Probir Mitro, Md. Sabuj Rana, Zabed Mazumder, Tajul Islam Mamun, Milton Roy, Md. Khademul Islam, Ashraful Alam Emon, Asikur Rahman, Md. Mukter Hossain

**Affiliations:** ^1^ Faculty of Veterinary, Animal and Biomedical Sciences, Sylhet Agricultural University, Sylhet, Bangladesh, sau.ac.bd; ^2^ Department of Medicine, Sylhet Agricultural University, Sylhet, Bangladesh, sau.ac.bd; ^3^ Department of Epidemiology and Public Health, Sylhet Agricultural University, Sylhet, Bangladesh, sau.ac.bd; ^4^ Department of Biomedical and Diagnostic Science, College of Veterinary Medicine, The University of Tennessee, Knoxville, Tennessee, USA, tennessee.edu

**Keywords:** antimicrobial resistance, Bangladesh, enterococci, human–animal–environment interface, meta-analysis, prevalence

## Abstract

**Background:**

Enterococci are important opportunistic pathogens responsible for a wide range of infections in humans and animals. Their ability to acquire and disseminate antimicrobial resistance (AMR) poses a growing public health concern. This study is aimed at estimating the pooled prevalence of enterococci and their AMR profiles among isolates from humans, animals, and environmental sources in Bangladesh.

**Methods:**

We conducted a systematic literature search in PubMed, Google Scholar, and CAB Abstracts in accordance with the Preferred Reporting Items for Systematic Reviews and Meta‐Analyses (PRISMA) guidelines. Studies published between January 2016 and November 2025 that reported the prevalence and/or AMR patterns of enterococci in Bangladesh were eligible for inclusion. A total of 36 studies met the inclusion criteria and were included in the final analysis. Pooled prevalence estimates were calculated using random‐effects meta‐analysis, with heterogeneity assessed via Cochran′s *Q* and the *I* ^2^ statistic; AMR proportions were estimated using the Wilson method, and publication bias was evaluated using Egger′s test and funnel plots.

**Results:**

The pooled prevalence of enterococci was estimated at 12.05% (95% CI: 7.10–19.70), with substantial heterogeneity (*I* ^2^ = 99.4*%*). Among eight identified *Enterococcus* spp., *Enterococcus faecalis* was the most prevalent (25.1%; 95% CI: 20.9–29.2), followed by *Enterococcus faecium* (6.3%; 95% CI: 4.6–7.9). High resistance rates were observed against cefuroxime (77.1%) and ceftriaxone (73.4%), whereas resistance to vancomycin was 20.9%.

**Conclusions:**

The findings underscore the urgent need for rational antimicrobial use, strengthened infection prevention and control measures, improved environmental hygiene, and integrated One Health surveillance across human, animal, and environmental settings to mitigate the burden of antimicrobial‐resistant enterococci.

## 1. Introduction

*Enterococcus*, a genus within the family Enterococcaceae, comprises zoonotic opportunistic pathogens that are capable of causing infections in both humans and animals [1]. These organisms are now widely recognized as important emerging bacteria due to their broad ecological adaptability, ranging from intestinal commensals to persistent environmental survivors and significant nosocomial pathogens with a strong capacity for multidrug resistance [2, 3]. Their ability to persist in soil, water, food, and wastewater highlights their role as indicators of fecal contamination. In addition, birds, fish, wild animals, and other mammals serve as important reservoirs, facilitating transmission through contaminated food or direct contact [4].

This Gram‐positive bacterial genus comprises more than 50 species, of which *Enterococcus faecalis* and *Enterococcus faecium* are the most prevalent in humans and animals, accounting for approximately 80%–90% of all clinical enterococcal infections [5, 6]. Other *Enterococcus* species such as *E. gallinarum*, *E. casseliflavus*, *E. durans*, *E. avium*, and *E. hirae* are infrequent and collectively represent less than 5% of clinical enterococcal isolates [7]. Enterococci are responsible for various healthcare‐associated infections in humans such as bloodstream and urinary tract infections, meningitis, neonatal sepsis, and various intra‐abdominal and pelvic infections [5, 8]. In animals, particularly livestock and poultry, enterococci are associated with septicemia, skeletal infections, mastitis, urinary tract infections, endocarditis, and other inflammatory conditions [9, 10]. Collectively, these infections pose substantial challenges to food safety and public health. According to the World Health Organization (WHO), mortality rates associated with enterococcal infections range from 14.3% to 32.3% [11]. The global prevalence of enterococcal infections continues to increase, accompanied by rising mortality. Concurrently, antimicrobial resistance (AMR) among enterococci has emerged as a critical public health concern due to their exceptional ability to acquire resistance genes and act as reservoirs of transferable AMR determinants [12, 13].

The overuse and misuse of antimicrobials in clinical medicine, veterinary practice, and agricultural production systems have significantly contributed to the emergence and dissemination of AMR enterococci in food animals and animal‐derived products [5, 14]. Sustained selective pressure has promoted resistance to multiple antimicrobial classes, including aminoglycosides, tetracyclines, chloramphenicol, penicillin, ampicillin, and glycopeptides [15]. Vancomycin was once considered a critical last‐line therapy for infections caused by multidrug‐resistant (MDR) organisms, including ampicillin‐resistant enterococci [16]. However, enterococci have increasingly acquired vancomycin resistance mediated by van gene‐associated elements, complicating treatment options and representing a growing global public health threat [5, 17]. Antibiotic resistance in enterococci shows marked regional variation, with the highest resistance reported in the Western Pacific region (23.1%) and the lowest in Europe (9.3%). Urinary tract infections are particularly affected, with resistance detected in approximately 37.4% of cases [18].

Although the global importance of addressing AMR is increasingly recognized, evidence on the transmission dynamics of *Enterococcus* across human, animal, and environmental interfaces remains limited, particularly in developing countries [19]. In Bangladesh, *Enterococcus* species are frequently detected in poultry farms, livestock production systems, hospital wastewater, surface water, human clinical samples, raw foods, and retail meat [20–22]. From a public health perspective, this widespread detection of *Enterococcus* underscores its significance as both an environmental contaminant and a reservoir of AMR genes [23]. However, data on the prevalence and diversity of enterococci, particularly *E. faecalis* and *E. faecium*, remain limited in Bangladesh [24]. A major challenge in Bangladesh is the frequent use of antibiotics in the absence of adequate regulatory oversight. This practice facilitates inappropriate prescribing by untrained individuals and promotes self‐medication, which are key drivers of increasing AMR [25]. According to the National Antimicrobial Resistance Surveillance, Bangladesh has experienced an 11% increase in antibiotic resistance over the past 5 years [26].

Enterococcal infections impose a substantial economic burden on both the healthcare system and the livestock sector in Bangladesh. In human healthcare settings, enterococci are increasingly associated with prolonged hospital stays, higher diagnostic costs, and increased expenditure on antimicrobial therapy [27]. In veterinary and agricultural contexts, enterococcal infections result in economic losses due to reduced productivity, increased treatment costs, and culling of affected animals [4]. Although comprehensive national mortality data are lacking, hospital‐based studies indicate that enterococci are among the leading causes of urinary tract infections in Bangladesh [27, 28]. Nevertheless, available data on human clinical cases remain insufficient to guide effective prevention and control strategies [20, 29]. Consequently, implementation of a One Health approach is essential to understand the circulation of enterococci across human, animal, and environmental domains, address AMR, and identify intervention points to reduce transmission [30]. Despite increasing reports on the prevalence and AMR profiles of *Enterococcus* spp. from individual and localized studies, a comprehensive national‐level synthesis for Bangladesh is lacking. Therefore, this systematic review and meta‐analysis aimed to synthesize published evidence to provide a comprehensive assessment of enterococci prevalence, species distribution across sources, and AMR patterns in Bangladesh.

## 2. Materials and Methods

### 2.1. Study Design

A systematic review and meta‐analysis was carried out to estimate the prevalence and AMR patterns of *Enterococcus* in Bangladesh. The study was conducted according to the Preferred Reporting Items for Systematic Reviews and Meta‐Analysis (PRISMA) checklist [31].

### 2.2. Search Strategy and Study Selection

A comprehensive literature search was performed in PubMed, Google Scholar, and CAB Abstracts to identify relevant studies published between January 2016 and November 2025. The search strategy combined keywords related to *Enterococcus* spp., antimicrobial or antibiotic resistance (including multidrug resistance and vancomycin‐resistant enterococci [VRE]), and prevalence, occurrence, or isolation, along with Bangladesh and One Health sources (human, animal, and environment). The full search strategy and database‐specific terms are provided in Table S2.

### 2.3. Study Eligibility Criteria

Studies were included if they (i) were conducted in Bangladesh, (ii) were published between 2016 and November 2025, (iii) were written in English, and (iv) reported data on the prevalence and/or AMR profiles of *Enterococcus* spp. Exclusion criteria included duplicate publications, noncomparable or incomplete data, absence of prevalence or AMR outcomes, and unavailability of full‐text articles. All retrieved records were exported to Mendeley Desktop (Version 1.19.5), and duplicate records were removed. Two reviewers independently screened titles and abstracts against the predefined eligibility criteria. Full‐text articles were then independently assessed by two additional reviewers. Disagreements were resolved by a third reviewer through discussion and consensus. The primary outcomes were (i) the prevalence of *Enterococcus* spp. and (ii) their AMR patterns. Prevalence was defined as the proportion of *Enterococcus*‐positive samples relative to the total number of samples tested in each study.

### 2.4. Data Extraction

Data were extracted independently by five authors using a standardized data extraction form. Extracted variables included study title, authors, year of publication, study region, study period, sample source (human, animal, and environment), diagnostic methods, *Enterococcus* spp., total sample size, number of positive isolates, antimicrobial agents tested, and resistance outcomes. Extracted data were cross‐checked, and discrepancies were resolved through verification by the primary author.

### 2.5. Quality Assessment

Quality assessment of studies was performed according to the modified Critical Appraisal Checklist recommended by the Joanna Briggs Institute (JBI) [32]. The checklist is composed of nine questions addressed by reviewers for each study. Each “yes” response was given a score of 1. Thus, final scores for each study could range from 0 to 9. Articles were classified as low (0–3), moderate (4–6), or high quality (7–9), and only high‐quality studies were analyzed [18, 23, 33].

Case definition: *Enterococcus* positivity was defined as the detection or isolation of *Enterococcus* spp. from human, animal, or environmental samples using culture alone or in combination with biochemical and/or molecular methods (e.g., PCR), as reported in the original studies. Samples identified as positive by any of these methods were considered *Enterococcus*‐positive for the meta‐analysis.

### 2.6. Statistical Analysis

The R packages “meta” [34] and “metafor” [35] were employed to estimate the models for meta‐analysis and visualize the results. In the primary analysis, the pooled prevalence of enterococci was estimated using both fixed‐ and random‐effects models, which account for within‐study variance alone and for both within‐ and between‐study variance, respectively. The prevalences were reported along with the 95% confidence intervals [36]. Estimation of the models was carried out using the Der‐Simonian–Laird random‐effects method [37]. For variables or outcomes reported by only one study, meta‐analysis was not performed due to insufficient data for quantitative pooling; instead, the reported prevalence was presented descriptively. The percentage of AMR was calculated as the number of resistant antimicrobials divided by the total number of antimicrobials tested, with corresponding confidence intervals estimated using the Wilson method [38]. These AMR summaries therefore represent aggregate resistance proportions rather than random‐effects pooled study‐specific estimates.

### 2.7. Heterogeneity Analysis

Heterogeneity among studies was assessed using Cochran′s *Q* statistic [39] and quantified with the *I* ^2^ statistic [40] to estimate the proportion of total variation attributable to differences between studies rather than to within‐study variability (chance). Heterogeneity was considered statistically significant when Cochran′s *Q* test produced a *p* value < 0.05, and the *I* ^2^ statistic exceeded 50%, consistent with commonly accepted thresholds where *I* ^2^ values of 25%, 50%, and 75% indicate low, moderate, and high heterogeneity, respectively [41]. The true between‐study variance (*τ* ^2^) and its standard deviation (*τ*) were estimated using the tau statistic to evaluate the degree of heterogeneity. Subgroup analyses were conducted to identify potential sources of heterogeneity in *Enterococcus* spp. prevalence. This analysis employed mixed‐effects models, with random‐effects models used to aggregate study effects within each subgroup and fixed‐effects models applied to determine whether there were significant differences between subgroups. Common between‐study variance was assumed across subgroups, and the within‐group estimates of *τ* ^2^ were pooled. The considered moderators included diagnostic methods, year of publication (2016–2019, 2020–2022, and 2023–2025), and source of infection (human, animal, and environment).

### 2.8. Publication Bias and Visualization

Forest plots were generated to illustrate heterogeneity in prevalence estimates and their corresponding 95% confidence intervals across studies. Therefore, Egger′s test and funnel plots were used to explore small‐study effects and potential publication bias [42]. All statistical analyses were conducted using R Programming (Version 4.4.2) [34].

## 3. Results

### 3.1. Search Results

**Figure 1:**
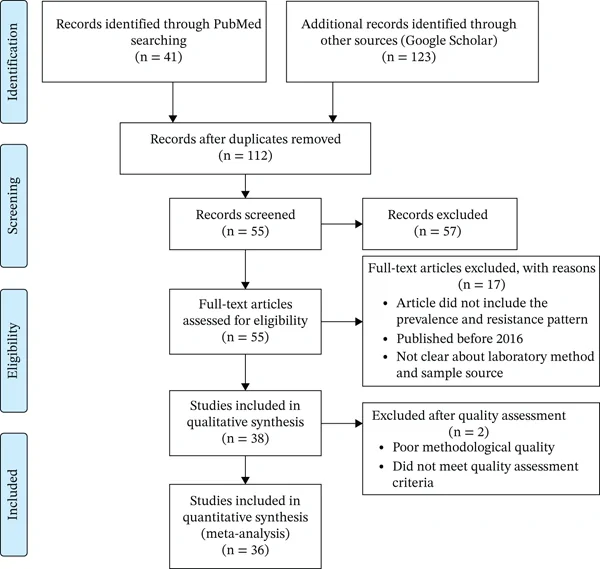
PRISMA flow diagram showing the literature search and study‐selection process.

The database and gray literature searches identified 164 documents. After removing duplicates, 112 records remained for title/abstract screening. Of these, 57 records were excluded, and 55 full‐text articles were assessed for eligibility. Following full‐text assessment, 17 articles were excluded for not meeting the predefined eligibility criteria, leaving 38 studies for qualitative synthesis. Subsequently, two studies were excluded based on the predefined quality assessment criteria. Finally, 36 studies were included for data extraction and meta‐analysis (Table S1, Figure 1).

### 3.2. Prevalence of Enterococci

**Figure 2:**
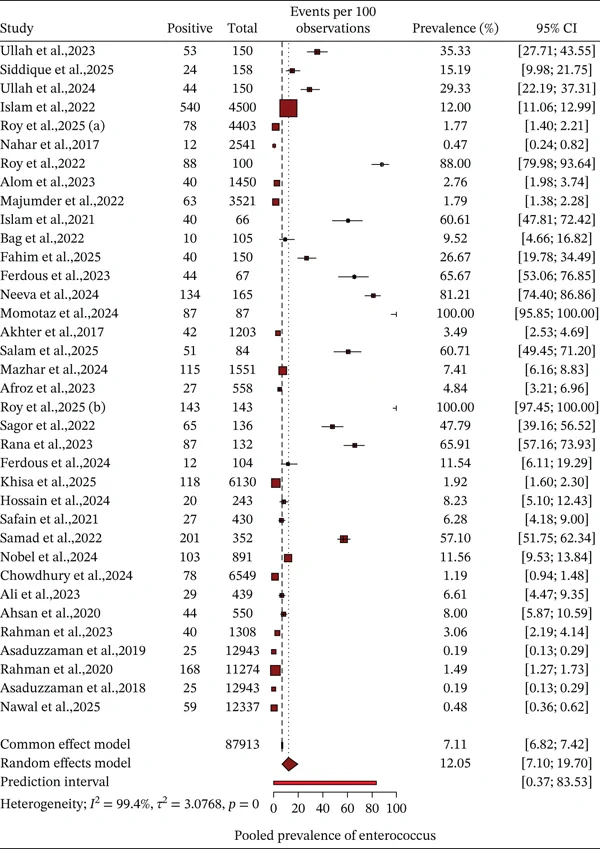
Forest plot of meta‐analysis showing pooled prevalence of *Enterococcus* spp. in studies conducted in Bangladesh.

The prevalence distributions of Enterococci in different categories are depicted in Table 1. A total of 36 articles from Bangladesh are presented on the prevalence of Enterococci. The overall pooled prevalence of Enterococci was estimated at 12.05% (95% CI: 7.10–19.70) (Figure 2). A significant moderator effect was observed for diagnostic method (QM = 37.6, *p* < 0.0001), indicating that differences in diagnostic approaches substantially influenced reported prevalence estimates (Table 1). The markedly highest prevalence estimates for the diagnostic method integrating culture, biochemical test, and PCR reported 59.7% (95% CI: 35.8–83.7; *I* ^2^ = 99.8*%*), whereas those employing only culture and biochemical methods yielded a low pooled prevalence of 4.0% (95% CI: 3.3–4.6; *I* ^2^ = 99.1*%*). Publication year, on the other hand, also showed a significant moderating effect (QM = 9.8, *p* = 0.0073) on Enterococci, accounting for 19.9% (*R* ^2^) of the true heterogeneity (Table 1). The pooled prevalence increased from 0.4% (95% CI: 0.2–0.7; *I* ^2^ = 92.3*%*) during 2016–2019 to 25.4% (95% CI: 21.1–29.8) in 2020–2022, and further to 28.6% (95% CI: 23.1–34.2) in 2023–2025. Based on the source of the sample, we categorized the prevalence of enterococci into human, animal, and environment. The highest pooled prevalence was found in the animal source (45.0%, 95% CI: 29.0–61.0, *I* ^2^ = 97.9*%*), followed by the environmental source (44.3%, 95% CI: 12.6–76.1, *I* ^2^ = 95.3*%*) and human origin (14.9%, 95% CI: 12.7–17.0; *I* ^2^ = 99.9*%*) (Table 1).

**Table 1:** Pooled prevalence of *Enterococcus* in Bangladesh based on different categories.

Variable	Level	No. of study	Sample tested			Heterogeneity							
			*N*	*n*	% (95% CI)	*Q* value	*p*	*I* ^2^ (%)	*τ* ^2^	*H* ^2^	QM	*R* ^2^ (%)	*p*
Diagnosis	Culture	5	8420	231	5.2 (3.1–7.3)	124.2	< 0.0001	91.2	3.1	149.3	37.6	52.2	< 0.0001
	Culture and biochemical	17	77,156	1502	4.0 (3.3–4.6)	2313.4	0	99.1					
	Culture, biochemical test, PCR	12	2082	997	59.7 (35.8–83.7)	396.0	0	99.8					
	Culture, PCR	2	255	50	18.0 (1.2–34.8)	10.6	0.0004	91.9					

Publication year	2016–2019	4	29,630	104	0.4 (0.2–0.7)	193.6	< 0.0001	92.3	5.1	238.9	9.8	19.9	0.0073
	2020–2022	10	21,034	1246	25.4 (21.1–29.8)	1866.0	0	99.5					
	2023–2025	22	37,249	1430	28.6 (23.1–34.2)	2616.3	0	99.9					

Sources	Human	24	86,263	2029	14.9 (12.7–17.0)	2755.9	0	99.9	4.4	213.2	12.8	29.3	0.0017
	Animal	11	1446	662	45.0 (29.0–61.0)	219.9	< 0.0001	97.9					
	Environment	2	204	85	44.3 (12.6–76.1)	20.4	< 0.0001	95.3					

Abbreviations: *τ* ^2^ (tau squared), between‐study variance—estimated variance of the distribution of true prevalence in the population of *Enterococcus*; CI, confidence interval; df, degrees of freedom; *H* ^2^, unaccounted variability (sampling variability)—the ratio of the standard deviation of the estimated overall prevalence from the random‐effects model compared with the standard deviation from the fixed‐effects model; *I* ^2^ (%), residual heterogeneity (unaccounted heterogeneity); *N*, total sample; *n*, number of positive sample; QM, coefficient of test for heterogeneity between subgroups; *R* ^2^, proportion of between‐study variance explained.

### 3.3. Pooled Prevalence and Distribution of *Enterococcus* Serotypes

*E. faecalis* showed the highest prevalence of 25.1% (95% CI: 20.9–29.2), followed by *E. faecium* 6.3% (95% CI: 4.6–7.9). The pooled prevalence of nonspecific enterococci was 6.3 (95% CI: 5.5–7.1) in the population. Other species, such as *E. hirae*, *E. raffinosus*, *E. avium*, *E. casseliflavus*, *E. durans*, and *E. gallinarum*, demonstrated a markedly low prevalence (< 1%). The prevalence of *Enterococcus* varies considerably between different sources. In human cases, *E. faecalis* was observed at 20.3% followed by nonspecific *Enterococcus* spp. (2.8%). Considering animal sources, nonspecific *Enterococcus* spp. was observed to have the highest prevalence of 65.1%, followed by *E. faecalis* (32.3%). *E. faecium* was highly prevalent in the environment (38.1%), followed by *E. faecalis* (25.8%) (Table 2).

**Table 2:** Source‐specific pooled prevalence of *Enterococcus* serotype.

Species	No. of study	Overall prevalence % (95% CI)	*Q* value	*I* ^2^ (%)	Source‐specific prevalence % (95% CI)		
					Human	Animal	Environment
*Enterococcus faecalis*	20	25.1 (20.9–29.2)	1479.4	99.4	20.3 (15.1–25.5)	32.3 (19.1–45.4)	25.8 (19.5–32.1)
*Enterococcus faecium*	11	6.3 (4.6–7.9)	689.2	96.5	1.0 (0.3–1.8)	28.0 (10.6–45.3)	38.1 (27.3–48.9)
*Enterococcus hirae*	2	0.13 (0.00–0.30)	2.7	0	0.13 (0.00–0.30)	—	—
*Enterococcus raffinosus*	2	0.23 (0.00–0.49)	7.4	19.5	0.23 (0.00–0.49)	—	—
*Enterococcus avium*	2	0.08 (0.00–0.18)	0.0	0	0.08 (0.00–0.18)	—	—
*Enterococcus casseliflavus*	1	0.07 (0.01–0.38)	0.0	0	0.07 (0.00–0.20)	—	—
*Enterococcus durans*	1	0.07 (0.00–0.20)	0.0	0	0.07 (0.00–0.20)	—	—
*Enterococcus gallinarum*	1	0.14 (0.04–0.50)	0.0	0	0.14 (0.00–0.33)	—	—
Nonspecific *Enterococcus* spp.	21	6.3 (5.5–7.1)	3881.4	99.2	2.8 (2.2–3.3)	65.1 (49.9–80.2)	—

### 3.4. Antibiotic Resistance Pattern of Enterococci

In the overall *Enterococcus* isolates, the highest antibiotic resistance rate was against cefuroxime with 77.1% (95% CI: 73.3–80.5), followed by ceftriaxone with 73.4% (95% CI: 69.8–76.8) and streptomycin 68.4% (95% CI: 59.4–76.2), whereas fosfomycin with 2.8% (95% CI: 1.7–4.8) had the lowest antibiotic resistance rate. This study investigated the AMR rates of enterococci among different sources (Table 3). Among human isolates, the highest resistance rate was observed for ceftriaxone at 77.7% (95% CI: 73.9–81.0), whereas vancomycin showed a resistance rate of 19.9% (95% CI: 17.5–22.4). Fosfomycin showed a low resistance rate of 0.9% (95% CI: 0.2–3.1), whereas no resistance to tigecycline was observed in this group. Notably, enterococci exhibited a high resistance rate of 90.0% (95% CI: 85.5–93.3) against rifampicin in animal sources, whereas vancomycin was 20.6% (95% CI: 17.2–24.4). The lowest was observed against fosfomycin (4.8%; 95% CI: 2.7–8.3). In environmental sources, resistance peaked for ampicillin at 76.0% (95% CI: 65.2–84.2), rifampicin at 53.3% (95% CI: 42.2–64.2), and erythromycin at 52.0% (95% CI: 40.9–62.9).

**Table 3:** Source‐specific resistance percentage of *Enterococcus* spp. against the most used antibiotics.

Antibiotics	Overall			% (95% CI) total		
	*N*	*n*	% (95% CI)	Human	Animal	Environment
Vancomycin	1573	328	20.9 (18.9–22.9)	19.9 (17.5–22.4) 1012	20.6 (17.2–24.4) 486	36.0 (26.1–47.3) 75
Penicillin	912	449	49.2 (46.0–52.5)	30.5 (26.7–34.7) 511	73.1 (68.5–77.2) 401	—
Ampicillin	994	408	41.0 (38.0–44.1)	22.8 (19.7–26.2) 654	76.2 (70.7–81.0) 265	76.0 (65.2–84.2) 75
Amoxicillin	695	228	32.8 (29.4–36.4)	29.4 (26.2–32.8) 666	33.8 (24.0–45.1) 29	—
Imipenem	785	234	29.8 (26.7–33.1)	31.4 (28.0–35.0) 711	65.5 (47.3–80.1) 74	—
Gentamicin	1161	446	38.4 (35.7–41.2)	40.2 (37.3–43.2) 1047	21.9 (15.3–30.4) 114	—
Streptomycin	114	78	68.4 (59.4–76.2)	—	68.4 (59.4–76.2) 114	—
Ciprofloxacin	1804	807	44.8 (42.5–47.1)	57.3 (54.5–59.9) 1243	16.9 (13.8–20.5) 486	17.3 (10.4–27.4) 75
Levofloxacin	1221	488	40.0 (37.3–42.7)	52.4 (49.1–55.7) 891	5.1 (3.0–8.5) 255	10.7 (5.5–19.7) 75
Tigecycline	119	3	2.5 (0.9–7.2)	0.0 (0.0–3.9) 95	12.5 (4.3–31.0) 24	—
Tetracycline	960	491	51.1 (48.0–54.3)	69.7 (64.9–74.0) 389	40.7 (36.5–45.1) 496	24.0 (15.8–34.8) 75
Doxycycline	496	266	53.6 (49.2–58.0)	53.6 (49.2–58.0) 496	—	—
Chloramphenicol	650	67	10.3 (8.2–12.9)	18.0 (11.4–27.2) 89	7.8 (5.7–10.6) 486	17.3 (10.4–27.4) 75
Erythromycin	922	561	60.8 (57.7–63.9)	57.6 (52.7–62.3) 401	65.2 (60.7–69.5) 446	52.0 (40.9–62.9) 75
Fosfomycin	459	13	2.8 (1.7–4.8)	0.9 (0.2–3.1) 228	4.8 (2.7–8.3) 231	—
Linezolid	1575	492	31.2 (29.0–33.6)	27.9 (25.4–30.7) 1099	41.1 (36.4–46.0) 401	26.7 (18.0–37.6) 75
Nitrofurantoin	1191	192	16.1 (14.1–18.3)	21.6 (18.9–24.7) 790	5.2 (3.5–7.9) 401	—
Rifampicin	476	282	59.2 (54.8–63.6)	20.0 (14.7–26.6) 170	90.0 (85.5–93.3) 231	53.3 (42.2–64.2) 75
Trimethoprim–sulfamethoxazole	268	139	51.9 (45.9–57.8)	51.9 (45.9–57.8) 268	—	—
Azithromycin	717	348	48.5 (44.9–52.2)	48.7 (45.0–52.3) 707	40.0 (16.8–68.7) 10	—
Cefuroxime	511	394	77.1 (73.3–80.5)	77.4 (73.5–80.9) 487	70.8 (50.8–85.1) 24	—
Meropenem	707	277	39.2 (35.6–42.8)	38.5 (34.9–42.3) 667	50.0 (35.2–64.8) 40	—
Ceftriaxone	609	447	73.4 (69.8–76.8)	77.7 (73.9–81.0) 524	47.1 (36.8–57.6) 85	—
Cefixime	529	277	52.4 (48.1–56.6)	51.7 (47.2–56.1) 484	60.0 (45.5–73.0) 45	—

Abbreviations: CI, confidence interval; *N*, total sample; *n*, number of resistant samples.

Among *Enterococcus* serotypes, the resistance rate of *E. faecalis* was highest against streptomycin (96.6%), cefuroxime (91.1%), and azithromycin (63.7%). A high resistance to azithromycin was reached at 85.71% in *E. faecium*, followed by cefuroxime (80.00%), penicillin (72.2%), and ampicillin (68.9%). The nonspecific isolates exhibited the most alarming pattern, with exceptionally high resistance to trimethoprim–sulfamethoxazole (90.80%) and cefuroxime (69.9%). Additionally, moderate to high resistance rates to penicillin (37.21%) and amoxicillin (37.3%) were observed in nonspecific *Enterococcus* spp. (Table 4).

**Table 4:** Antimicrobial resistance pattern of *Enterococcus* serotypes against different antibiotics.

Antibiotics	% (95% CI) total		
	*Enterococcus faecalis*	*Enterococcus faecium*	Nonspecific *Enterococcus*
Vancomycin	11.1 (9.1–13.6) 756	25.3 (20.2–31.3) 233	31.7 (28.1–35.6) 583
Penicillin	49.4 (45.3–53.5) 573	72.2 (63.4–79.5) 115	37.2 (31.1–43.7) 223
Ampicillin	43.1 (39.0–47.2) 557	68.9 (60.6–76.2) 132	25.3 (20.8–30.5) 304
Amoxicillin	16.1 (12.5–20.4) 336	51.1 (41.1–60.9) 94	37.3 (32.4–42.4) 354
Imipenem	59.8 (53.8–65.4) 271	41.9 (26.4–59.2) 31	13.5 (10.5–17.2) 393
Gentamicin	31.1 (26.9–35.6) 434	53.8 (43.7–63.5) 93	45.5 (40.8–50.3) 422
Streptomycin	96.6 (82.8–99.4) 29	66.7 (52.1–78.6) 45	50.0 (35.2–64.8) 40
Ciprofloxacin	44.5 (40.9–48.1) 724	34.5 (28.6–40.9) 226	47.7 (44.3–51.0) 854
Levofloxacin	25.1 (21.8–28.8) 577	26.7 (19.5–35.4) 116	59.2 (55.0–63.3) 527
Tigecycline	12.5 (4.3–31.0) 24	—	0.0 (0.0–3.9) 95
Tetracycline	53.4 (49.3–57.3) 594	45.9 (38.9–53.1) 185	49.2 (42.0–56.4) 181
Doxycycline	53.1 (44.5–61.4) 130	46.9 (30.9–63.6) 32	54.5 (49.1–59.8) 334
Chloramphenicol	9.4 (6.8–12.7) 384	10.4 (6.6–16.0) 164	13.7 (8.4–21.7) 102
Erythromycin	66.9 (62.7–70.8) 519	72.5 (65.4–78.7) 171	39.0 (32.9–45.4) 231
Fosfomycin	1.3 (0.6–3.1) 375	9.6 (5.0–17.9) 83	—
Linezolid	31.0 (27.9–34.2) 817	26.6 (20.3–34.0) 158	32.9 (29.2–36.7) 599
Nitrofurantoin	14.8 (12.3–17.8) 654	18.1 (12.8–25.1) 149	17.5 (14.1–21.6) 388
Rifampicin	55.3 (50.2–60.2) 380	75.8 (66.3–83.3) 95	—
Trimethoprim–sulfamethoxazole	29.6 (23.1–37.1) 162	9.1 (2.5–27.8) 22	90.8 (83.5–95.1) 98
Azithromycin	63.7 (57.4–69.6) 237	85.7 (65.4–95.0) 21	39.0 (34.6–43.5) 459
Cefuroxime	91.1 (85.9–94.5) 169	80.0 (49.0–94.3) 10	69.9 (64.7–74.6) 332
Meropenem	21.0 (15.9–27.2) 200	47.6 (28.3–67.6) 21	46.3 (41.9–50.7) 486
Ceftriaxone	89.7 (83.6–93.6) 145	89.1 (78.2–94.9) 55	65.5 (60.8–70.0) 409
Cefixime	43.7 (34.5–53.3) 103	60.0 (45.5–73.0) 45	53.8 (48.8–58.7) 381

Abbreviations: CI, confidence interval

### 3.5. Publication Bias

**Figure 3:**
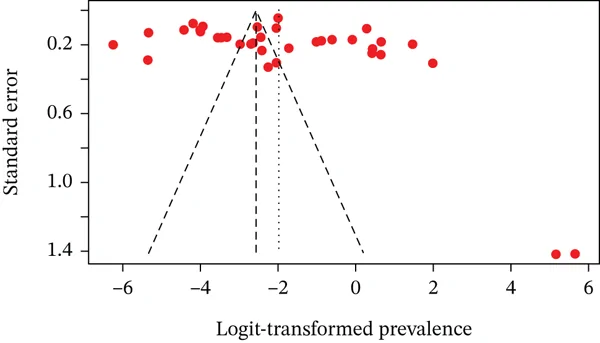
Funnel plot of meta‐analysis showing publication bias in studies reporting the prevalence of *Enterococcus* spp.

The funnel plot exhibited some degree of asymmetry, indicating a potential publication bias present in this meta‐analysis (Figure 3). However, Egger′s test did not indicate statistically significant funnel‐plot asymmetry (*p* = 0.36), although the possibility of publication bias cannot be entirely excluded. The contour funnel indicates a substantial contribution of the studies to the overall meta‐analysis at different levels of significance. Therefore, these findings should be interpreted with caution.

## 4. Discussion

AMR is a major health concern in livestock and human health that affects and causes a heavy burden on low‐ and middle‐income countries globally, which is also an increasing threat to high‐income countries. Antibiotic‐resistant bacterial infections caused an estimated 1.2 million deaths in 2019, more deaths than AIDS or malaria [43]. This systematic review and meta‐analysis provides a comprehensive and robust analysis of the current studies conducted from 2016 to 2025 on the prevalence of *Enterococcus* in humans, animals, and the environment, as well as its pooled AMR profile in Bangladesh.

The pooled prevalence of *Enterococcus* spp. was estimated at 12.05% in Bangladesh. The present study findings are relatively comparable with the study conducted in Southeast Asia (India, Nepal, and Vietnam), where the pooled prevalence range was found between 10% and 30% [44–46]. In contrast, the prevalence and resistance rates of *Enterococcus* spp. reported in high‐income countries such as Europe and North America have been comparatively lower than the findings from low‐ and middle‐income countries [47].

Although the overall pooled estimate across all sample sources was moderate, substantial differences were observed across source categories. The prevalence was substantially higher in animals (45.0%) and the environmental sample (44.3%) compared with humans (14.9%). These differences should not be interpreted solely as reflecting true biological risk because the underlying studies differed in populations, sampling frames, specimen types, diagnostic approaches, and study settings. Nevertheless, the ability of *Enterococcus* spp. to persist in soil and water and to disseminate between hosts and environmental reservoirs may contribute to circulation across One Health interfaces [48]. Moreover, inadequate veterinary healthcare, monitoring, and regulatory services, intervention of excessive informal animal health service providers, and farmers′ knowledge gap on drugs and AMR recorded in Bangladesh may be the potential cause of higher prevalence in animals [25].

Among different *Enterococcus* serotypes, *E. faecalis* showed a higher pooled prevalence (25.1%) than *E. faecium* (6.3%). This distribution is consistent with global evidence identifying *E. faecalis* as the predominant clinical and commensal species, whereas *E. faecium* is more frequently associated with elevated AMR and persistence in hospital and environmental settings. Similarly, species‐specific patterns have been reported in Europe and North America, where *E. faecium* has increasingly adapted to healthcare‐associated niches and exhibits higher resistance profiles than *E. faecalis* [47]. In Bangladesh, widespread and often unregulated antibiotic use in livestock and aquaculture, together with inadequate waste management practices, may contribute to sustained environmental contamination and increased opportunities for human exposure to resistant *Enterococcus* strains [25].

In our study, we found alarmingly high resistance rates to key antimicrobial classes. Overall, resistance to cephalosporins, including cefuroxime (77.1%) and ceftriaxone (73.4%), was pervasive. These high resistance rates are consistent with the well‐established intrinsic nonsusceptibility of *Enterococcus* to cephalosporins, which is associated with the low‐affinity penicillin‐binding proteins involved in cell‐wall synthesis. However, the high resistance observed in our study may also reflect the broader selective pressure associated with antimicrobial use in clinical and agricultural settings, including empirical antibiotic use, over‐the‐counter availability, and extensive use in livestock production [49, 50]. Similar high resistance levels have been reported from other low‐ and middle‐income countries from clinical enterococcal isolates, such as Pakistan [51]. High resistance to streptomycin (68.4%) further erodes the utility of traditional combination therapies. Human isolates exhibited high resistance to cephalosporins, whereas notable resistance to vancomycin (19.9%) signals the emergence of VRE, a recognized global threat. VRE prevalence has been reported across food chains and clinical settings worldwide, with several studies demonstrating their presence in nonclinical reservoirs, including food animals and environmental waters [52]. On the other hand, species‐specific AMR profiles revealed that *E. faecalis* exhibited extremely high resistance to streptomycin and cephalosporins, and moderate resistance to penicillin and ciprofloxacin. Identification of carbapenem resistance among *E. faecalis* further signals the emergence of MDR lineages that could complicate empiric therapy. In *E. faecium*, a similarly concerning resistance profile was observed, including high rates of macrolide and aminoglycoside resistance. These patterns reflect global concerns wherein *E. faecium* often harbors greater resistance than *E. faecalis*, particularly to ampicillin, gentamicin, and newer agents, as documented in numerous international meta‐analyses [18]. Nonspecific enterococcal isolates demonstrated high resistance to trimethoprim–sulfamethoxazole, emphasizing the importance of species identification in AMR surveillance. Lumping diverse species into a nonspecific category may obscure reservoirs of resistance genes capable of horizontal transfer.

A major strength of this study is that, to the best of our knowledge, it is the first systematic review and meta‐analysis to integrate human, animal, and environmental data on *Enterococcus* spp. prevalence and AMR in Bangladesh, providing a comprehensive One Health perspective. It offers a broader overview of species distribution and AMR patterns and identifies important variations across different sources, which may support future surveillance and research in Bangladesh. Nevertheless, several limitations must be acknowledged. Extreme between‐study heterogeneity was observed, with *I* ^2^ approaching or exceeding 95%–99% in many overall and subgroup analyses. Although diagnostic method, publication period, and source were statistically significant moderators, these factors explained only part of the observed variability. Additional heterogeneity may have resulted from differences in study populations, sampling strategies, specimen types, diagnostic methods, antimicrobial susceptibility testing methods and interpretive criteria, and study periods. Regarding the study period, we included studies published within the predefined period for which eligible evidence was available; earlier studies meeting our criteria were not identified. For AMR, resistance estimates were calculated descriptively, but variations in AST methods and interpretive criteria may have affected the comparability of resistance estimates across studies [51]. In addition, inconsistent antimicrobial susceptibility testing standards and limited molecular characterization restricted deeper inference regarding resistance mechanisms and transmission pathways. Future research should prioritize standardized surveillance protocols and molecular characterization of resistance determinants to better understand transmission dynamics across One Health domains. Strengthening antimicrobial stewardship in both human and veterinary sectors, improving environmental waste management, and expanding integrated AMR surveillance systems are critical steps to mitigate the growing burden of antimicrobial‐resistant enterococci in Bangladesh.

## 5. Conclusion

This systematic review and meta‐analysis estimated an overall pooled prevalence of *Enterococcus* spp. of 12.1% across the included human, animal, and environmental sources in Bangladesh between 2016 and 2025. Among the identified species, *E. faecalis* and *E. faecium* were the most prevalent across human, animal, and environmental sources. High resistance rates were observed against cefuroxime, ceftriaxone, streptomycin, erythromycin, rifampicin, and trimethoprim–sulfamethoxazole, highlighting substantial therapeutic challenges. The findings underscore the urgent need for rational antimicrobial use, strengthened infection prevention and control measures, improved environmental hygiene, and integrated One Health surveillance in human, animal, and environmental settings to mitigate the burden of antimicrobial‐resistant enterococci.

## Author Contributions

Md. Abdur Nur Sakib: final approval of manuscripts, writing—review and editing, writing—original draft, visualization, validation, software, methodology, formal analysis, data curation, and conceptualization. Probir Mitro: final approval of manuscripts, writing—review and editing, writing—original draft, methodology, and data curation. Md. Sabuj Rana: final approval of manuscripts, writing—review and editing, writing—original draft, methodology, data curation, and conceptualization. Zabed Mazumder: final approval of manuscripts, writing—review and editing, writing—original draft, methodology, formal analysis, and data curation. Tajul Islam Mamun: final approval of manuscripts, writing—review and editing, writing—original draft, validation, and methodology. Milton Roy: final approval of manuscripts, methodology, writing—review and editing, writing—original draft, and formal analysis. Md. Khademul Islam: final approval of manuscripts, writing—review and editing, writing—original draft, and formal analysis. Ashraful Alam Emon: final approval of manuscripts, writing—review and editing, writing—original draft, software, methodology, and data curation. Asikur Rahman: final approval of manuscripts, methodology, writing—review and editing, writing—original draft, and formal analysis. Md. Mukter Hossain: writing—review and editing, writing—original draft, visualization, validation, supervision, software, resources, project administration, methodology, investigation, funding acquisition, formal analysis, and conceptualization.

## Funding

No funding was received for this manuscript.

## Conflicts of Interest

The authors declare no conflicts of interest.

## Supporting information


**Supporting Information** Additional supporting information can be found online in the Supporting Information section. Supporting Material S1 (.docx): PRISMA 2020 checklist. Supporting Material S2 (.docx): Table S1: Characteristics of 36 included studies. Table S2: Database and search terms used.

## Data Availability

The data that support the findings of this study are available from the corresponding author upon reasonable request.
