# Supplementary material for: Prevalence and Antimicrobial Resistance Profiles of Enterococci Isolated From Humans, Animals, and Environment in Bangladesh: A Systematic Review and Meta‐Analysis
Source: Int J Microbiol. 2026 Sep 23;2026:6574310. doi: 10.1155/ijm/6574310 (PMC13599370; doi:10.1155/ijm/6574310)
Supplement: Supplementary file 1 [file IJM-2026-6574310-s001.zip › Updated_Supplementary Materials 2.docx]

**Table S1:** Characteristics of 36 included studies.

| **Study** | **Study Period** | **Division** | **Source** | **Diagnosis** | **Species** | **N** | **n** | **AMR Pathogen** | **Antibiotics (Included)** | **JBI Score** |
| --- | --- | --- | --- | --- | --- | --- | --- | --- | --- | --- |
| Ullah et al.,2023[1] | January 2022 – March 2023 | Chattogram | Animal | Culture, Biochemical, PCR | *E. faecium* | 150 | 53 | *E. faecium* | VA, P, AMP, CIP, LEV, NOR, TE, E, LZD, C, FOS, NIT, RIF | 9 |
| Siddique et al.,2025[2] | October 2023 – June 2024 | Dhaka and Barisal | Animal | Culture | *E. faecalis* | 158 | 24 | *E. faecalis* | VA, P, AMP, CXM, CIP, LEV, TGC, TE, E, LZD, C, NIT | 9 |
| Ullah et al.,2024[3] | October 2023 – June 2024 | Chattogram | Animal | Culture, Biochemical, PCR | *E. faecalis* | 150 | 44 | *E. faecalis* | VA, P, AMP, CIP, LEV, NOR, TE, E, LZD, C, NIT, FOS, FIF | 9 |
| Islam et al.,2022[4] | 1st September 2016 – 30th November 2018 | Dhaka | Human | Culture and Biochemical | *E. faecalis* | 4,500 | 540 | *E. faecalis* | N/A | 9 |
| Roy et al.,2025(a)  [5] | January 2017 and February 2020 | Mymensingh | Human | Culture and Biochemical | *E. spp.* | 4403 | 78 | *E. spp.* | P, GEM, CIP, TE, AZM, LZD, NIT | 9 |
| Nahar et  al.,2017[6] | January –June 2015 | Dhaka | Human | Culture and Biochemical | *E. spp.* | 2541 | 12 | *E. spp.* | VA, AMX, CEX, CTR, IMP, MEM, CIP, L ZD | 7 |
| Roy et al.,2022[7] | July 2019 –March 2021 | Mymensingh | Animal | Culture and Biochemical | *E. spp.* | 100 | 88 | *E. faecium* | VA, TEI, CFX, CTR, IMPP, GEN, S, CIP, NOR, TE, E, C | 9 |
|  |  |  |  |  | *E. faecium* | 100 | 45 |  |  |  |
| Alom et al.,2023[8] | March 2022 –March 2023 | Rangpur | Human | Culture | *E. spp.* | 1,450 | 40 | *E. spp.* | VA, AMX, CTR, IMP, GEM, CIP, LEV, DO, LZD, C, NIT | 8 |
|  |  |  |  |  | *E. faecalis* |  | 9 |  |  |  |
|  |  |  |  |  | *E. faecium* |  | 7 |  |  |  |
|  |  |  |  |  | *E. gallinarum* |  | 2 |  |  |  |
|  |  |  |  |  | *E. casseliflavus* |  | 1 |  |  |  |
|  |  |  |  |  | *E. avium* |  | 1 |  |  |  |
|  |  |  |  |  | *E. durans* |  | 1 |  |  |  |
|  |  |  |  |  | *2E. hirae* |  | 1 |  |  |  |
| Majumder et al.,2022[9] | June 2011–June 2021 | Chattogram | Human | Culture and Biochemical | *E. spp.* | 3521 | 63 | *E. spp.* | VA, AMP, AMX, CXM, CFX, CTR, IMP, MEM, GEM, CIP, LEV, TGC, DO, E, AZM, LZD, NIT | 9 |
| Islam et al.,2021[10] | November 2019 and November 2020. | Khulna | Animal | Culture, Biochemical, PCR | *E. spp.* | 66 | 40 | *E. spp.* | VA, AMP, MEM, GEN, S, CIP, TE, C | 7 |
| Bag et al.,2022[11] | January 2019 and June 2021 | Dhaka, Mymensingh, Rajshahi | Animal | Culture, PCR | *E. faecalis* | 105 | 10 | *E. faecalis* | TE, AZM | 8 |
| Fahim et al.,2025[12] | October 2023 and April 2024 | Mymensingh and Rajshahi | Animal  And Environment | Culture, PCR | *E. faecalis* | 150 | 40 | *E. faecalis* | VA, AMP, CIP, LEV, TE, LZD, C, RIF | 9 |
| Ferdous et al.,2023[13] | July 2022 –March 2023 | Chattogram, Khulna, Sylhet, and Dhaka | Animal | Culture, Biochemical, PCR | *E. spp* | 67 | 48 | *E. faecalis,*  *E. faecium* | VA, P, CIP, LEV, NOR, TE, E, LZD, C, NIP, FOS, RIF | 9 |
|  |  |  |  |  | *E. faecalis* |  | 44 |  |  |  |
|  |  |  |  |  | *E. faecium* |  | 15 |  |  |  |
| Neeva et al,.2024[14] | 1st July 2020 – 30th June 2021 | Sylhet | Human | Culture, Biochemical, PCR | *E.spp* | 165 | 23 | *E. faecalis,*  *Other spp.* | VA, CXM, CFX, CTR, MEM, GEM, CIP, AZM, LZD, NIT | 9 |
|  |  |  |  |  | *E. faecalis* |  | 16 |  |  |  |
| Momotaz et al,.2024[15] | March 2019 –February 2020 | Dhaka | Human | Culture, Biochemical test, PCR | *E. raffinosus* | 87 | 2 | *E. faecalis,*  *E. faecium* | VA, AMP, GEM, CIP, QD, NIT | 8 |
|  |  |  |  |  | *E. faecalis* |  | 65 |  |  |  |
|  |  |  |  |  | *E. faecium* |  | 20 |  |  |  |
| Akhter et al,.2017[16] | January 2010 –December 2010 | Dhaka | Human | Culture and Biochemical | *E. raffinosus* | 1203 | 1 | *E. faecalis,*  *E. faecium* | VA, AMX, CXM, CTR, IMP, GEM, CIP, NIT | 8 |
|  |  |  |  |  | *E. faecalis* |  | 42 |  |  |  |
|  |  |  |  |  | *E. faecium* |  | 10 |  |  |  |
|  |  |  |  |  | *E. avium* |  | 1 |  |  |  |
| Salam et al,.2025[17] | N/A | Mymensingh | Environment | Culture, Biochemical test, PCR | *E. faecium* | 84 | 32 | *E. faecalis,*  *E. faecium* | VA, AMP, CIP, LEV, TE, E, LZD, C, RIF | 8 |
|  |  |  |  |  | *E. faecalis* |  | 19 |  |  |  |
| Mazhar et al,.2024[18] | January 2023 –October 2023 | Dhaka | Human | Culture and Biochemical | *E. spp.* | 1551 | 115 | *E. spp.* | VA, P, AMP, CIP, LEV, TE, LZD, NIT | 9 |
| AFROZ et al,.2023[19] | N/A | Dhaka | Human | Culture and Biochemical | *E. spp.* | 558 | 27 | *E. spp.* | AMP, AMX, CXM, CFX, CTR, MEM, GEN, LEV, DO | 7 |
| Roy et al,.2025(b)  [20] | February 2023 –January 2024 | Mymensingh, | Human | Culture, Biochemical test, PCR | *E. faecalis* | 143 | 135 | *E. faecalis,*  *E. faecium,*  *E.hirae* | VA, TEI, P, AMP, IMP, GEN, LEV, TEC, E, LZD, SXT, FOS, RIF | 9 |
|  |  |  |  |  | *E. faecium* |  | 7 |  |  |  |
|  |  |  |  |  | *E. hirae* |  | 1 |  |  |  |
| Sagor et al,.2022[21] | N\A | Dhaka | Animal | Culture, Biochemical test, PCR | *E. spp.* | 136 | 65 | *E. faecalis* | VA, P,  AMP, AMX, IMP, GEM, CIP, TE, E, LZD, C, NIT | 8 |
|  |  |  |  |  | *E. faecalis* |  | 29 |  |  |  |
| Rana et al,.2023[22] | October 2021 – December 2022, | Mymensingh | Animal | Culture, Biochemical test, PCR | *E. faecalis* | 132 | 87 | *E. faecalis* | VA, P, AMP, CIP, LEV, NOR, TE, E, LZD, C, NIT, FOS, RIF | 9 |
| Ferdous et al.,2024[23] | January 2023 –April 2023 | Dhaka | Human | Culture and Biochemical | *E. spp.* | 104 | 12 | *E. spp.* | VA, TEI, AMX, CFX, CTR, MEM, GEN, CIP, LEV, TGC, TEC, DO, LZD, | 7 |
| Khisa et al,.2025[24] | December 2022 to May 2023 | Dhaka | Human | Culture | *E. faecalis* | 6130 | 97 | *E. faecalis,*  *E. faecium* | VA, P, AMP, AMX, IMP, MEM, CIP, LEV, TE, AZM, LZD, NIT | 8 |
|  |  |  |  |  | *E. faecium* |  | 21 |  |  |  |
| Hossain et al,.2024[25] | January 2019 –December 2020 | Dhaka | Human | Culture | *E. spp.* | 243 | 20 | *E. spp.* | AMX, CXM, CEX, CTR, IMP, GEM, CIP, LEV, NOR, TGC, DO, AZM, SXT, | 9 |
| Safain et al,.2021[26] | 2015 to 2019 | Dhaka | Human | Culture and Biochemical | *E. faecalis* | 430 | 27 | *E. faecalis* | VA, P, GEN, CIP, TE, E, AZM, LZD, C, SXT, RIF | 9 |
| Samad et al,.2022[27] | 2016 to 2017 | Dhaka | Animal | Culture, Biochemical test, PCR | *E. faecalis* | 352 | 115 | *E. spp* | VA, P, CIP, TE, E, LZD, C, NIT | 9 |
|  |  |  |  |  | *E. faecium* |  | 36 |  |  |  |
|  |  |  |  |  | *E. spp.* |  | 201 |  |  |  |
| Nobel et al,.2024[28] | January 2018 –March 2022 | Dhaka | Human | Culture and Biochemical | *E. faecalis* | 891 | 103 | *E. faecalis* | ANX, CXM, CFX, CTR, MEM, GEN, CIP, LEV, TE, AZM, LZD, NIT | 8 |
| Chowdhury et al,. 2024[29] | from January 2018 –December 2018 | Dhaka | Human | Culture and Biochemical | *E. spp.* | 6549 | 78 | *E. spp.* | CXM, CFX, CIP, LEV, SXT | 9 |
| Ali et al,. 2023[30] | March 2021 –December 2021 | Dhaka | Human | Culture | *E. spp.* | 439 | 29 | *E. spp* | VA, P, AMX, CTR, IMP, GEN, CIP, LEV, DO, AZM, LZD | 7 |
| Ahsan et al,. 2020[31] | January 2017 –December 2017 | Dhaka | Human | Culture, Biochemical test, PCR | *E. faecalis* | 550 | 33 | *E. faecalis,*  *E. faecium* | VA, TEI, AMP, IMP, GEN, CIP, DO, LZD, NIT | 7 |
|  |  |  |  |  | *E. faecium* |  | 11 |  |  |  |
| Rahman et al,. 2023[32] | November 2021 –April 2022. | Dhaka | Human | Culture and Biochemical | *E. spp.* | 1308 | 40 | *E. spp.* | CXM, CFX, CTR, MEM, GEN, CIP, LEV, DO, AZM | 8 |
| Asaduzzaman et al,. 2019[33] | 1st January 2016 –31st December | Dhaka | Human | Culture and Biochemical | *E. spp.* | 12943 | 25 | *E. spp.* | GEN | 8 |
| Rahman et al,. 2020[34] | January 2018 –December 2019 | Dhaka | Human | Culture and Biochemical | *E. spp.* | 11,274 | 168 | *E. spp.* | VA, AMX, IMP, MEM, GEM, CIP, E, AZM, LZD | 7 |
| Asaduzzaman et al,. 2018[35] | 1st January 2016 –31st December, 2016 | Dhaka | Human | Culture and Biochemical | *E. spp.* | 12943 | 25 | *E. spp.* | CFX | 7 |
| Nawal et al,. 2025[36] | January –December, 2022 | Dhaka | Human | Culture and Biochemical | *E. spp.* | 12337 | 59 | *E. spp.* | VA, P, AMP, CIP, LZD, NIT | 9 |

VA = Vancomycin; P = Penicillin; AMP = Ampicillin; AMX = Amoxycillin; IMP = Imipenem; GEN = Gentamicin; S = Streptomycin; CIP = Ciprofloxacin; LEV = Levofloxacin; TGC = Tigecycline; TE = Tetracycline; DO = Doxycycline; C = Chloramphenicol; E = Erythromycin; FOS = Fosfomycin; LZD = Linezolid; NIT = Nitrofurantoin; RD = Rifampicin; SXT = Trimethoprim–Sulfamethoxazole; AZM = Azithromycin; CXM = Cefuroxime; MEM = Meropenem; CTR = Ceftriaxone; CFX = Cefixime.

**Table S2.** Database and search terms used

| **Database** | **Search term** | **Number of studies** |
| --- | --- | --- |
| **PubMed** | (("Enterococcus" OR Enterococcus OR Enterococci OR "Enterococcus faecalis" OR "Enterococcus faecium") AND ("Drug Resistance, Bacterial"[MeSH Terms] OR "antimicrobial resistance" OR "antibiotic resistance" OR "multidrug resistance" OR MDR OR "vancomycin resistant enterococci"  OR VRE) AND (prevalence OR occurrence OR distribution OR isolation) AND ("Bangladesh"  OR Bangladesh)) | 41 |
| **Google Scholar** | "Enterococcus" OR "Enterococci" OR "Enterococcus faecalis" OR "Enterococcus faecium" AND "antimicrobial resistance" OR "antibiotic resistance" OR "multidrug resistance" OR MDR OR VRE AND prevalence OR occurrence OR isolation OR "antibiotic susceptibility" AND human OR clinical OR patient OR animal OR poultry OR livestock OR cattle OR environment OR water OR food AND “Bangladesh” | 123 |

**Reference**

1.	Ullah MA, Islam MS, Rana ML, Ferdous FB, Neloy FH, Firdous Z, Hassan J, Rahman MT. Resistance Profiles and Virulence Determinants in Biofilm-Forming *Enterococcus faecium* Isolated from Raw Seafood in Bangladesh. Pathogens. 2023 Aug 28;12(9):1101. doi: 10.3390/pathogens12091101.

2.	Siddique, A.B., Ahmed, S., Shahadat, M.N. *et al.* Uncovering antimicrobial resistance patterns in *Pseudomonas aeruginosa* and *Enterococcus faecalis* isolated from farm animals in Bangladesh. *Discov Bact* 2, 7 (2025). https://doi.org/10.1007/s44351-025-00018-6

3.	Ullah MA, Islam MS, Ferdous FB, Rana ML, Hassan J, Rahman MT. Assessment of prevalence, antibiotic resistance, and virulence profiles of biofilm-forming *Enterococcus faecalis* isolated from raw seafood in Bangladesh. Heliyon. 2024 Oct 11;10(20):e39294. 10.1016/j.heliyon.2024.e39294.

4.	Islam MA, Islam MR, Khan R, Amin MB, Rahman M, Hossain MI, Ahmed D, Asaduzzaman M, Riley LW. Prevalence, etiology and antibiotic resistance patterns of community-acquired urinary tract infections in Dhaka, Bangladesh. PLoS One. 2022 Sep 15;17(9):e0274423. doi: 10.1371/journal.pone.0274423.

5.	Roy S, Barua HT, Sujan MJ, Hossain A, Paul SK, Nasreen SA, Ahmed S, Haque N, Habib ZH, Rahman A, et al. Trends and patterns of antimicrobial resistance in a tertiary care hospital in Bangladesh. Microbiol Res. 2025;16(2):30. doi:10.3390/microbiolres16020030.

6.	Nahar A, Hasnat S, Akhter H, Begum N. Evaluation of antimicrobial resistance pattern of uropathogens in a tertiary care hospital in Dhaka city, Bangladesh. South East Asia J Public Health. 2018;7(2):12–18. doi:10.3329/seajph.v7i2.38851.

7.	Roy K, Islam MS, Paul A, Ievy S, Talukder M, Sobur MA, Ballah FM, Khan MSR, Rahman MT. Molecular detection and antibiotyping of multi-drug resistant *Enterococcus faecium* from healthy broiler chickens in Bangladesh. Vet Med Sci. 2022;8(1):200–210. doi:10.1002/vms3.669.

8.	Alam MS, Zakaria M, Rahman R. Examining antimicrobial resistance in *Enterococcus* species: a single-center cross-sectional study. Saudi J Pathol Microbiol. 2023;8(10):257–262. doi:10.36348/sjpm.2023.v08i10.004.

9.	Majumder MMI, Mahadi AR, Ahmed T, Ahmed M, Uddin MN, Alam MZ. Antibiotic resistance pattern of microorganisms causing urinary tract infection: a 10-year comparative analysis in a tertiary care hospital of Bangladesh. Antimicrob Resist Infect Control. 2022 Dec 10;11(1):156. doi: 10.1186/s13756-022-01197-6.

10.	Islam MS, Paul A, Talukder M, Roy K, Sobur MA, Ievy S, Nayeem MMH, Rahman S, Nazir KHMNH, Hossain MT, Rahman MT. Migratory birds travelling to Bangladesh are potential carriers of multi-drug resistant *Enterococcus* spp., *Salmonella* spp., and *Vibrio* spp. Saudi J Biol Sci. 2021;28(10):5963–5970. doi:10.1016/j.sjbs.2021.06.053.

11.	Bag MAS, Arif M, Riaz S, Khan MSR, Islam MS, Punom SA, Ali MW, Begum F, Islam MS, Rahman MT, Hassan J. Antimicrobial Resistance, Virulence Profiles, and Public Health Significance of *Enterococcus faecalis* Isolated from Clinical Mastitis of Cattle in Bangladesh. Biomed Res Int. 2022 Sep 27;2022:8101866. doi: 10.1155/2022/8101866.

12.	Fahim NAI, Rana ML, Hasan MAE, Salam S, Masud RI, Huda N, Saha S, Rahman MT. Biofilms and antibiotic resistance profile of Enterococcus faecalis in selected dairy cattle farm environments in Bangladesh. PLoS One. 2025 May 19;20(5):e0323667. doi: 10.1371/journal.pone.0323667.

13.	Ferdous FB, Islam MS, Ullah MA, Rana ML, Punom SA, Neloy FH, Chowdhury MNU, Hassan J, Siddique MP, Saha S, Rahman MT. Antimicrobial Resistance Profiles, Virulence Determinants, and Biofilm Formation in Enterococci Isolated from Rhesus Macaques (*Macaca mulatta*): A Potential Threat for Wildlife in Bangladesh? Animals (Basel). 2023 Jul 11;13(14):2268. doi: 10.3390/ani13142268.

14.	Neeva NI, Zafrin N, Jhuma AA, Chowdhury SK, Fatema K, Rifat TA. Antimicrobial susceptibility patterns of *Enterococcus* species and molecular detection of *Enterococcus faecalis* isolated from patients with urinary tract infection in a tertiary care hospital in Bangladesh. Indian J Microbiol. 2024;64(3):1025–1034. doi:10.1007/s12088-024-01216-7.

15.	Momotaz T, Khan RR, Afroz F, Chowdhury S, Islam MN, Sarwar MT, Sattar ANI. Phenotypic and genotypic virulence factors and their association with antibiotic resistance in clinical isolates of *Enterococcus* species in Bangladesh. Arch Microbiol Immunol. 2024;8:376–382. doi:10.26502/ami.936500184.

16.	Akhter J, Ahmed S, Anwar S. Antimicrobial susceptibility patterns of *Enterococcus* species isolated from urinary tract infections. Bangladesh J Med Microbiol. 2017;8(1):16–20. doi:10.3329/bjmm.v8i1.31069.

17.	Salam S, Fahim NA, Ashraf MN, Masud RI, Jahan R, Antor MT, Hasan MA, Bakhtiyar Z, Jany DA, Rana ML, Islam MS. Virulence profiling, biofilm formation, and antimicrobial resistance pattern of enterococci associated with poultry and livestock feeds. J Appl Poult Res. 2025:100590. doi:10.1016/j.japr.2025.100590.

18. Mazhar I, Alim R, Andalib S, Munwar S. Prevalence and antimicrobial susceptibility pattern of urinary *Enterococcus* isolates in a tertiary care hospital: prevalence and antimicrobial susceptibility of urinary *Enterococcus*. J Med Coll Women Hosp. 2024;20(2):37–43. doi:10.3329/jmcwh.v20i2.77295.

19.	Afroz T. Prevalence and anti-bio gram of *Enterococcus* species in different samples: a cross-sectional study. Research Square [Preprint]. 2023 Nov 21. doi:10.21203/rs.3.rs-3633503/v1.

20.	Roy S, Aung MS, Paul SK, Khan MNA, Nasreen SA, Hasan MS, Haque N, Barman TK, Khanam J, Sathi FA, et al. Isolation of vanA-mediated vancomycin-resistant *Enterococcus faecalis* (ST1912/CC116) and *Enterococcus faecium* (ST80/CC17), optrA-positive linezolid-resistant *E. faecalis* (ST32, ST1902) from human clinical specimens in Bangladesh. Antibiotics (Basel). 2025;14(3):261. doi:10.3390/antibiotics14030261.

21.	Sagor MS, Hossain MS, Islam T, Mahmud MA, Miah MS, Karim MR, Giasuddin M, Samad MA. Phenotypic and genotypic antibiotic resistance and virulence profiling of *Enterococcus faecalis* isolated from poultry at two major districts in Bangladesh. Pak Vet J. 2022;42(2):153–160. doi:10.29261/pakvetj/2022.019.

22.	Rana ML, Firdous Z, Ferdous FB, Ullah MA, Siddique MP, Rahman MT. Antimicrobial Resistance, Biofilm Formation, and Virulence Determinants in *Enterococcus faecalis* Isolated from Cultured and Wild Fish. Antibiotics (Basel). 2023 Aug 28;12(9):1375. doi: 10.3390/antibiotics12091375.

23.	Ferdous M, Jabin T, Islam S, Sarker MMR, Rahman S, Esrat A, Mahmud A, Hossain MI, Alam ST, Uddin MA. Current status of drug-resistant patterns of Gram-positive clinical isolates collected from renowned diagnostic centers of Dhaka, Bangladesh. Biomed Biotechnol Res J. 2024;8(1):53–59. doi:10.4103/bbrj.bbrj_290_23.

24.	Khisa S, Khan AA, Chowdhury JP, Rahman W. Prevalence and antibiotic sensitivity pattern of enterococci causing urinary tract infection in a tertiary care hospital of Bangladesh. J Armed Forces Med Coll, Bangladesh. 2025;20(2):27–29. doi:10.3329/jafmc.v20i2.80407.

25.	Hossain MJ, Azad AK, Shahid MSB, Shahjahan M, Ferdous J. Prevalence, antibiotic resistance pattern for bacteriuria from patients with urinary tract infections. Health Sci Rep. 2024 Apr 11;7(4):e2039. doi: 10.1002/hsr2.2039.

26.	Safain KS, Bhuyan GS, Hasib SH, Islam MS, Mahmud-Un-Nabi MA, Sultana R, Tasnim S, Noor FA, Sarker SK, Islam MT, Rahat A, Leung DT, Domman D, Manzoor F, Anwar S, Bhuiyan MA, Chowdhury EK, Qadri SS, Qadri F, Mannoor K. Genotypic and phenotypic profiles of antibiotic-resistant bacteria isolated from hospitalised patients in Bangladesh. Trop Med Int Health. 2021;26(7):720–729. doi:10.1111/tmi.13584.

27.	Samad MA, Sagor MS, Hossain MS, Karim MR, Mahmud MA, Sarker MS, Shownaw FA, Mia Z, Card RM, Agunos A, Johanna L. High prevalence of vancomycin non-susceptible and multi-drug resistant enterococci in farmed animals and fresh retail meats in Bangladesh. Vet Res Commun. 2022;46(3):811–822. doi:10.1007/s11259-022-09906-7.

28.	Nobel FA, Ahammad H, Saha SR, Asaduzzaman M, Nashra A, Jebin RA, Zahan Y, Kamruzzaman M, Babu G, Islam MJ. Determination of multi-drug resistance profile of isolated Gram-positive and Gram-negative bacteria from clinical pus samples in Bangladesh. J Adv Biotechnol Exp Ther. 2024;7(1):178–189. doi:10.5455/jabet.2024.d16.

29.	Chowdhury SS, Tahsin P, Xu Y, Mosaddek ASM, Muhamadali H, Goodacre R. Trends in Antimicrobial Resistance of Uropathogens Isolated from Urinary Tract Infections in a Tertiary Care Hospital in Dhaka, Bangladesh. Antibiotics (Basel). 2024 Sep 27;13(10):925. doi: 10.3390/antibiotics13100925.

30.	Islam S, Rahman MA, Rahaman S. Prevalence and Antibiotic Susceptibility Pattern of Enterococcus Spp. Isolated from Urine Samples. Molecular Mechanism Research. 2023.

31.	Ahsan TA, Hemel MM, Shamsuzzaman SM. Prevalence of high-level gentamicin resistance and distribution of its genes in Enterococcus species isolated from patients of urinary tract infection in a Tertiary Care hospital in Bangladesh. Asian J Microbiol Biotechnol Environ Sci. 2020;22:174-80.

32.	Rahman B, Islam MR, Sultana R, Alam ST, Mehjabin T, Haque BN, Moniruzzaman SM, Hossain MI, Rashna SN, Uddin MA. Determination of the antibiotic susceptibility pattern of Gram-positive bacteria causing urinary tract infection in Dhaka, Bangladesh. Biomed Biotechnol Res J. 2023;7(3):432–437. doi:10.4103/bbrj.bbrj_159_23.

33.	Asaduzzaman M, Juliana FM, Islam A, Kumar A, Jafor MA, Hosen ME, Alam MJ, Hossain N, Das B, Zaman NR, Arbia L. Evaluation of Gentamicin's Antimicrobial Resistance Pattern Against Uropathogens in a Tertiary Health Care Center in Dhaka City, Bangladesh. Evaluation. 2019;67.

34.	Rahman F, Akhter H, Hasnat S, Begum N. Prevalence and antimicrobial sensitivity profile of uropathogens in a tertiary care hospital of Dhaka city. Bangladesh J Med Microbiol. 2020;14(2):3–11. doi:10.3329/bjmm.v14i2.57792.

35.	Asaduzzaman M, Shamim A, Mian MS, Alam MJ, Juliana FM, Hossain N, Das B, Asma R, Morshed M, Mitra S. Resistance pattern of cefixime against uropathogens causing urinary tract infection in selected areas of Dhaka city, Bangladesh. Int J Eng Sci. 2018;7(1):33–39. doi:10.9790/1813-0701013339.

36.	Nawal N, Begum MF, Nasrin M, Rahman F, Karim R, Alam M, Bhuiyan MM, Sujan MJ. Bacteriological profile and antibiotic susceptibility pattern of clinical isolates from a tertiary care hospital in Dhaka, Bangladesh. Fortune J Health Sci. 2025;8:667–674. doi:10.26502/fjhs.320.
